# Growth of Executive Functions in Preschool-Age Children During the COVID-19 Lockdown: Empirical Evidence

**DOI:** 10.11621/pir.2022.0209

**Published:** 2022-06-30

**Authors:** Elena A. Chichinina, Margarita N. Gavrilova

**Affiliations:** a Lomonosov Moscow State University, Moscow, Russia

**Keywords:** Working memory, cognitive flexibility, inhibition, development, COVID-19, lockdown, social restrictions

## Abstract

**Background:**

During the lockdown for COVID-19, children were limited in a number of activities which are essential for the development of executive functions (play, social interaction, and organized sport). Earlier studies found an increase in executive function issues in children during the pandemic, based on caregivers’ reports.

**Objective:**

The present study was a pioneer in exploring the dynamics of children’s executive function development during the lockdown. Our purpose was to explore the effect of the lockdown on the growth of executive functions in children over a one-year period, as compared to their peers before the pandemic.

**Design:**

The sample consisted of two cohorts of children. All the children had been attending the same kindergartens but in different periods of time. The executive functions of both groups were assessed twice, with a year’s break in-between (the first group was assessed before the pandemic; the second, during the pandemic). These groups were comparable in gender composition, age, and family’s place of residence.

**Results:**

The results have confirmed concerns about the slower growth of executive functioning in children during the lockdown versus their peers before the pandemic, especially for cognitive flexibility and working memory. Inhibition was not significantly affected by the lockdown. Moderation analysis showed that the lockdown impacted girls differently than boys in terms of working memory. The negative effect of social restrictions on working memory was significantly higher in females.

**Conclusion:**

Our findings illuminate the negative effects the pandemic-related social restrictions had on the growth of children’s cognitive flexibility and working memory. For working memory, the effect of social isolation varied depending on the child’s gender.

## Introduction

Executive function is an umbrella term for cognitive processes which are necessary for problem solving and goal-oriented behavior (Friedman & Miyake, 2017; Karbach & Kray, 2016). Numerous studies have shown the importance of executive functions as a predictor for language development, numeric skills, self-regulation, communication, transition to school, and academic achievement (Barenberg et al., 2011; Bellon et al., 2019; Gashaj et al., 2019; Kovyazina et al., 2021). Longitudinal studies have confirmed that the development of executive functions in preschool age significantly determines personal achievement and life satisfaction even into adulthood (Poon, 2018). For decades, such findings have invited clinical and research attention to exploring and elaborating ways to train children’s executive functions (Bergman Nutley et al., 2008; Diamond, 2012; Thibodeau et al., 2016).

Executive functioning is studied in a range of scientific traditions (Snyder et al., 2015). The diversity of approach is due to the complexity and multidimensionality of this construct, which includes biological, psychological, and developmental aspects (Anderson, 2001). The term “executive” is used in neuropsychology in studying the prefrontal cortex as the neurobiological and neuropsychological basis for goal-oriented behavior (Hillman et al., 2014; Nejati et al., 2018). The clinical perspective focuses on studying impairments of executive functions and their associations with various forms of psychopathology (Wante et al., 2017; Chutko et al., 2022).

Within the cognitive and developmental approaches, executive functioning is considered a psychological phenomenon. Cognitive psychology focuses on exploring the nature of executive functions as high-level cognitive processes (Snyder et al., 2015). Developmental psychology studies individual performance and maps its developmental trajectories (Bell et al., 2018; Friedman & Miyake, 2017). The Cultural-Historical Theory of Development (Vygotsky, 2004) and Bronfenbrenner’s Ecological Systems Theory (Bronfenbrenner, 1992) shifted developmental psychology’s perspective by bringing into focus the environmental and societal influences on child development in general, and on executive functioning in particular. These approaches aim to explore how the social, cultural, and historical environment can influence children’s developmental processes (Fleer et al., 2019; Veraksa et al., 2020). Activity Theory (Leontiev, 2005) is another important approach which emphasizes the child’s everyday experiences as a factor which could influence development.

### The Risks of COVID-19 lockdown for executive functions development

Influential developmental approaches (Cultural-Historical Theory of Development, Bronfenbrenner’s Ecological Systems Theory, and Activity Theory) suggest that, apart from neuropsychological and physiological factors, a child’s environment and everyday experiences play a significant role in executive function development (Fleer et al., 2017; Vidal Carulla et al., 2021). This view is supported by numerous empirical studies. Environmental characteristics partly explain deficits in executive functions among children (Doebel, 2020). Research shows that a child’s executive functions may be impacted by his/her family background and environment (Miller-Cotto et al., 2022; Leonova, 2020), as well as by the lack of developmental activities (Stucke et al., 2022). Play (Fleer et al., 2017, 2019; Thibodeau et al., 2016; Veraksa et al., 2020; Vidal Carulla et al., 2021), sport and physical activity (Gashaj et al., 2019; Jäger et al., 2016; Maurer & Roebers, 2019; Tvardovskaya at al., 2020), and interaction with peers and caregivers (Stucke et al., 2022; Gardiner & Iarocci, 2018) have beneficial effects on developing executive functions. By contrast, stress, social deprivation, and poor physical health, which were common phenomena during the lockdown, are harmful to executive functions (Diamond & Ling, 2016). During the COVID-19 lockdown, children experienced limitations on socializing with peers, play, and physical activity (skating, running, structured activities etc.).

The negative consequences of the lockdown on executive functioning have been documented in some recent studies. Navarro-Soria et al. (2021) found executive functioning deficits among children and adolescents diagnosed with ADHD. Polizzi et al. (2021) have shown that parents distressed by the lockdown reported that their children were having difficulties associated with deficits in executive functioning. However, some studies have shown that, in terms of the consequences of the lockdown on their developing executive functions, children of different ages were affected to different degrees (Hendry et al., 2022). In infants, the increase in time spent at home with their family has been shown to have had positive effects. Hendry and colleagues have explored the positive role of the home environment (SES, family activities) in the development of infants’ executive functions. Another study got similar results regarding speech development in children under 36 months of age during the lockdown (Kartushina, 2022), a result which may suggest that the severity of the lockdown consequences could depend on the child’s age.

### Kindergarten Operations and Social Restrictions in Russia

In Russia, preschool education is the first stage (from the ages of 3 to 7) of the state-funded general educational system. Public preschool institutions follow the guidelines of the State Standard for Early Childhood Care and Education (ECCE ) (Federal Law of the Russian Federation, 2012). According to the Standard, kindergartens provide not only childcare, but also a full educational program (including cognitive, arts, sports, and dance lessons for children). Although preschool education is not compulsory, over 96% of children age 3 to 7 years attend public kindergartens (Ministry of Education of the Russian Federation), normally five times a week. The majority of public preschool institutions are open 10–12 hours per day.

In the course of the pandemic, Russian kindergartens had to change their operations, as did preschool institutions all over the globe (Almondes et al., 2021). In the first pandemic wave (February 2020 — June 2020), a strict national lockdown was introduced as one of the measures of the COVID-19 containment policy. Use of public transport and moving around in general was allowed for professional or medical purposes only. Preschool institutions remained closed for eight months starting from March 2020. They were reopened in the central part of Russia (Official website of the Mayor of Moscow) during the second, the third, and the fourth waves (October 2020 to December 2021). This decision was based on the updated statistics that registered few to no cases of COVID-19 among preschoolers.

### Current Study

During the lockdown, children were limited in a number of the activities essential for executive functions development for eight months. The purpose of this study was to explore the effect of the lockdown on the growth of executive functions in children over a one-year period by comparing it to the dynamic in their peers before the pandemic, and to investigate whether the effect of social isolation on executive functions development varied depending on the child’s gender.

## Methods

### Participants

The data were collected in Moscow (Russia) from October 2017 to October 2020. The sample consisted of two cohorts of children. All the children had been attending the same kindergartens but in different periods of time. The first group (n = 298) was assessed before the pandemic: Time 1 = October 2017 (5 y.o); Time 2 = October 2018 (6 y.o.). The second group (n = 340) was assessed during the pandemic: Time 1 = October 2019 (5 y.o); Time 2 = October 2020 (6 y.o.). The groups were comparable in gender composition, and their families’ places of residence and age. The only difference between the samples was the time of the assessments: before or during the pandemic. Executive functions were assessed twice (with a year break inbetween) in both groups.

### Procedure

The executive function assessment was carried out individually by trained research assistants. The study was approved by the Ethics Committee of the Russian Psychological Society, and all parents gave their written informed consent for their children to participate in the study. All measures had previously been adapted and validated on a Russian monolingual sample and shown high psychometric qualities (Veraksa et al., 2020).

The *Dimensional Change Card Sort* (DCCS, Zelazo, 2006) was used to measure cognitive flexibility. In this task, the children are asked to sort, in three rounds and according to different rules, cards depicting colored objects. The task assesses the child’s ability to switch between the rules.

An *Inhibition* test (Korkman, Kirk & Kemp, 2007) was used to assess children’s ability to inhibit automatic cognitive responses. In the first part, the child is asked to name the shape or the direction of an arrow(s) (naming trials). In the second part, the child is asked to name the shape or direction conversely: *i.e.,* to say “circle” when a square is presented and vice versa (inhibition trials).

*Sentence Repetition* (Korkman, Kirk & Kemp, 2007) was used to assess verbal working memory. The test contained sentences of gradually increasing complexity. The child was asked to repeat each sentence after the tester who pronounced it in a neutral voice.

### Statistical analysis

An Independent Samples T-Test was used to verify the comparability of the two groups at Time 1 as to age, working memory, cognitive flexibility, and inhibition. For the main analysis, ANCOVA with two levels of the between-subjects factors of “Social restrictions” (“no restrictions” or “lockdown”) was used to explore the effect of the pandemic restrictions for each of the executive functions at the age of 6, when controlling for the individual variations assessed at the age of 5. Moderation analysis was run to explore whether child gender moderated the effect of social restrictions on executive function development. The level of significance was set at p < 0.05 for all the analyses. Partial eta square was reported as an estimation of effect size.

## Results

### Preliminary Analysis and Descriptive Statistics

As a first step, an Independent Samples T-Test was run to confirm the equality of the two groups at the baseline in terms of age and executive functions performance. There were no significant differences (p > .05) in the children’s ages at the baseline between the first (M = 5.28, SD = .09) and second groups (М = 5.29, SD = .11). Nor were significant differences found between the groups at the first assessment (at the age of 5) in cognitive flexibility, working memory, and inhibition (both speed of information processing and number of errors) (p > .05). But, one year later, differences in cognitive flexibility, working memory, and inhibition (time) were found to be significant (see *Table 1*). The children who had spent eight months of the year in lockdown showed significantly poorer results compared to the children who had spent the same period of time without any social restrictions. The number of errors in the inhibition task was the only variable that was not significantly different between two groups after one year.

**Table 1:** Descriptive statistics for executive functions among children at both assessments

	Social restrictions	5 y.o. assessment				6 y.o. assessment			
		M	SD	t	p	M	SD	t	p
Cognitive flexibility	No restrictions	18.71	2.74	1.06	0.287	20.77	2.54	–4.97	< .001
	Lockdown	18.93	2.52			19.74	2.72		
Working memory	No restrictions	17.60	4.07	–1.17	0.240	20.70	4.31	–4.11	< .001
	Lockdown	17.24	3.69			19.42	3.51		
Inhibition (time)	No restrictions	64.53	16.43	0.76	0.446	51.62	10.52	1.97	0.050
	Lockdown	65.59	16.38			53.59	13.12		
Inhibition (errors)	No restrictions	3.61	6.67	0.47	0.635	1.26	3.26	1.65	0.099
	Lockdown	3.86	5.98			1.73	3.50		

### Main Analysis

ANCOVA with two levels of the between-subjects factors of “Social restrictions” (“no restrictions” or “lockdown”) was used to explore the effect of the pandemic restrictions for each executive function at the age of 6 (see *Table 2*). The main effect of social restrictions was found to be significant for the growth of cognitive flexibility (F(1,639) = 32.92, p = .019, η^2^p = 0.049) and working memory (F(1,620) = 20.08, p < .001, η^2^p = 0.031). Improvements in the growth of both variables were less in children during the lockdown.

**Table 2:** Results of ANCOVA for the effects of social restrictions on executive function variables with assessment at age 5 y.o. as a co-variate

Variable	Effects	Sum of Squares	F	p	η^2^p
Cognitive flexibility	Cognitive flexibility (5 y.o.)	443.39	71.76	< .001	0.101
	Social restrictions	203.42	32.92	< .001	0.049
	Child gender	38.01	6.15	0.013	0.010
	Social restrictions ✳ Child gender	0.29	0.047	0.828	0.000
	Residuals	3941.84			
Working memory	Working memory (5 y.o.)	4113.4	504.90	< .001	0.449
	Social restrictions	163.6	20.08	< .001	0.031
	Child gender	88.4	10.85	0.001	0.017
	Social restrictions ✳ Child gender	39.6	4.86	0.028	0.008
	Residuals	5043.00			
Inhibition (time)	Inhibition (time) (5 y.o.)	70.12	1.74	0.187	0.003
	Social restrictions	2.98	0.07	0.786	0.000
	Child gender	41.69	1.04	0.309	0.002
	Social restrictions ✳ Child gender	0.08	0.01	0.965	0.000
	Residuals	22175.66			
Inhibition (errors)	Inhibition (errors) (5 y.o.)	83.91	7.47	0.006	0.013
	Social restrictions	25.09	2.23	0.136	0.004
	Child gender	0.23	0.02	0.887	0.000
	Social restrictions ✳ Child gender	1.85	0.16	0.685	0.000
	Residuals	6188.59			

*Note. Table shows the different dependent variables (executive functions), effects (social restriction condition and child gender), mean of squares (Type 3), F- and P-values, effect sizes (η^2^p). Degrees of freedom (df) = 1.*

**Figure 1.**
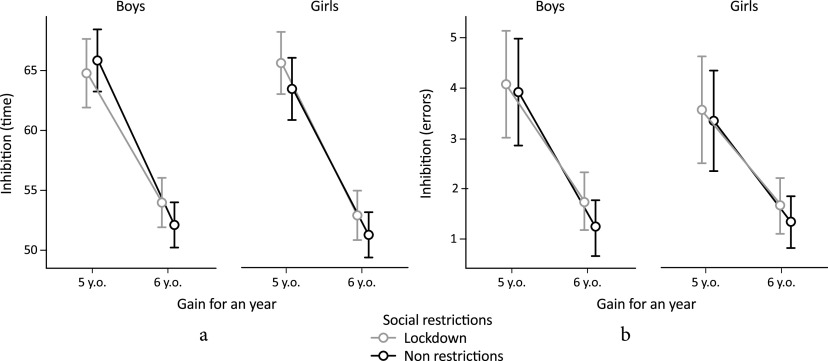
Growth of inhibitory control from 5 to 6 years old for boys and girls in two cohorts: (a) speed of information processing in seconds; (b) number of errors

No significant effect of social restrictions was found for inhibition (both speed of information processing and number of errors) (see *Figure 1*).

**Figure 2.**
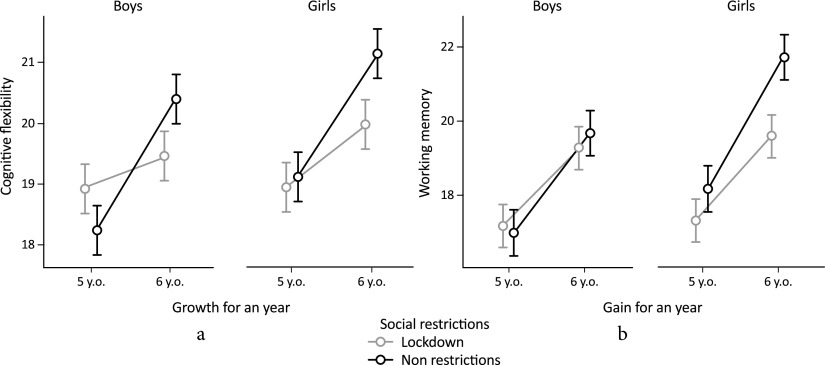
Graphic representation of the moderating effect of child gender on (a) cognitive flexibility and (b) working memory from 5 to 6 years of age during lockdown and no restrictions period

The results indicate that child gender also had a significant effect on cognitive flexibility (F(1,639) = 6.15, p = .013, η^2^p = 0.01) and working memory (F(1,639) = 10.85, p = .001, η^2^p = 0.017). The increase in both abilities over the one-year period was larger for girls after controlling for covariates (*Figure 2*). Additionally, given the gender-specific development of executive functions, it was pertinent to explore whether child gender moderates the relationship between social restrictions experiences and executive function growth. Based on the results obtained, analysis was conducted to check the potential moderating effects of child gender on cognitive flexibility and working memory. The moderating effect on the interaction between child gender and social restrictions was found to be significant for working memory (Z = 2.85, p > .004), but not for cognitive flexibility (Z = .466, 95% CI [.549, 2.96], p > .05).

To gain a better understanding of this result, a graphical representation of the moderation analysis was plotted (*Figure 2b*). The graph shows a positive slope for both genders, indicating that working memory had developed during the year in both the absence and presence of social restrictions. However, the negative effect of social restrictions on executive functions growth had been significantly greater in females. Interaction between social restrictions and child gender in the moderation analysis suggests that girls tend to show more negative effects on working memory under lockdown conditions.

## Discussion

During the lockdown for COVID-19, children were limited to a certain extent in a number of activities essential for executive function development. Research has suggested that lack of play (Fleer et al., 2017, 2019; Thibodeau et al., 2016; Veraksa et al., 2020; Vidal Carulla et al., 2021; Colliver et al., 2022), and of sport or physical activities (Gashaj et al., 2019; Jäger et al., 2016; Maurer & Roebers, 2019; Tvardovskaya at al., 2020) has a significant impact on executive functions among children. During the lockdown period, many of these activities were not available to the children in the usual amounts.

However, no empirical studies on the growth of executive functions in preschool-aged children during COVID-19 lockdown had been published before this study. The available research had examined the effect of the pandemic on infants or adolescents. Research findings have shown that while, for toddlers, the pandemic period had been a positive factor due to the large increase in child-parent interaction (Kartushina, 2022; Hendry et al., 2022), for older children the effect was adverse (Navarro-Soria et al., 2021; Polizzi et al., 2021).

The current study sought to examine the effect of lockdown on executive function growth among children over a one-year period by comparing it with the dynamic in their peers before the pandemic. An additional objective was to investigate whether child gender moderates the effect of social restrictions on executive functions. The present study is a natural experiment that would not have been possible outside the unprecedented pandemic situation. Previous research has documented the effectiveness of communication, play, sport or physical activities to promote executive functions development in children. However, it would be unethical to investigate how their omission (or reduction), would impact children’s growth of executive functions by deliberately depriving children of these activities.

The results have confirmed concerns about the slower growth of executive functions in children during the COVID-19 lockdown versus their peers before the pandemic. In particular, the growth in cognitive flexibility and working memory was less during lockdown. Inhibition (both speed of information processing and number of errors) was the only executive function that was not significantly affected by the lockdown. The increase in cognitive flexibility and working memory in one year was on average larger in girls, when controlling for individual variation at the baseline and social restrictions. Moderation analysis showed that lockdown impacted girls differently than boys in terms of working memory development. The negative effect of social restrictions on working memory was significantly higher in females.

An earlier study had found an increase in executive function issues in children during the pandemic based on caregivers’ reports (Navarro-Soria et al., 2021; Polizzi et al., 2021). To our knowledge, the present study is a pioneer in exploring the dynamic in children’s executive function development during the lockdown for COVID-19. However, the study findings are consistent with earlier research on the benefits of children’s activities such as play, communication, sports, and dance classes as executive function training (Diamond, 2012; Diamond & Ling, 2016; Vidal Carulla et al., 2021). As discussed above, the lack of these activities could be exactly the factor which had negatively influenced cognitive flexibility and working memory development during this period. In regard to working memory, this effect was particularly strong in girls.

In contrast, the present study has not obtained any evidence that inhibition had been affected by the lockdown. A possible explanation is that sustainable inhibition growth regardless of social restrictions and the child’s gender is a result of the other more relevant influences such as neuropsychological processes (Hillman et al., 2014; Nejati et al., 2018) or child–caregiver relationships (Stucke et al., 2022; Gardiner & Iarocci, 2018).

On a broader level, inhibition may be determined by common cultural context more than other executive functions. In contrast to working memory and cognitive flexibility, inhibitory control is constantly required for the child to meet the cultural expectations of adults. Both at home and in kindergarten, adults in Russia tend to limit their children’s spontaneous behavior and emotions from a young age (Veraksa et al., 2021). The cultural specificity of upbringing in this country is to manage the child’s behavior mainly through prohibitions (“don’t cry,” “don’t behave like that,” “don’t be like a baby”). Such a relationship probably creates a different ecology of childhood than in other cultures (Bronfenbrenner, 1992). Hence, the results of the current study regarding inhibitory control may be culturally specific and should therefore be interpreted with caution.

Overall, our findings point to the social importance of pre-school education for child development. An essential aspect of the lockdown was a lack of a structured and systematic educational process for children. As mentioned in the Introduction, public pre-school education provides not only childcare services, but also includes an educational component (cognitive, creative, and sporting activities) (Kalimullin et al., 2021). From a practical perspective, these results may implicitly support the importance of a systematic educational process for the sustainable development of children.

## Limitations

The findings of the present study should be examined in light of its limitations, which were primarily related to the available data. Although both cohorts of children attended the same kindergarten and lived in the same neighborhood, the lack of objective socio-demographic data means that assumptions of equality cannot be made. No data was collected on how the children spent the lockdown (the opportunities and activities available to the child could have varied from family to family). Also, no data have been collected on the family and cultural factors, which may also have influenced the results, and thus were not considered in this study.

## Conclusion

The results obtained confirmed concerns about the slower growth of executive functions in children during the lockdown. The growth in cognitive flexibility and working memory was less during the lockdown than in peers before the pandemic. For working memory, the effect of social isolation was moderated by the child’s gender. According to the results, inhibition was the only executive function that has not been significantly affected by the lockdown. The findings illuminate the effects of pandemic-related social restrictions on the development of executive functions in children. The children who participated in this study are involved in an ongoing longitudinal research project. In the near future there will be new data permitting evaluation of the long-term trajectory of executive function development among current study sample.
